# Home based telemedicine intervention for patients with uncontrolled hypertension: - a real life - non-randomized study

**DOI:** 10.1186/1472-6947-14-52

**Published:** 2014-06-12

**Authors:** Palmira Bernocchi, Simonetta Scalvini, Fabio Bertacchini, Francesca Rivadossi, Maria Lorenza Muiesan

**Affiliations:** 1 Fondazione Salvatore Maugeri, IRCCS. Telemedicine Service - Institute of Lumezzane, Brescia, Italy; 2 Department of Medical and Surgical Sciences, University of Brescia, Brescia, Italy; 3 Department of Cardiology, Fondazione Salvatore Maugeri, IRCCS. Institute of Lumezzane, Brescia, Italy

**Keywords:** Hypertension, Blood pressure, Telemedicine, Telemonitoring

## Abstract

**Background:**

Control of blood pressure is frequently inadequate in spite of availability of several classes of well tolerated and effective antihypertensive drugs. Several factors, including the use of suboptimal doses of drugs, inadequate or ineffective treatments and poor drug compliance may be the reason for this phenomenon. The aim of the current non- randomized study was to evaluate the effectiveness of a Home-Based Telemedicine service in patients with uncontrolled hypertension.

**Methods:**

74 patients were enrolled in a Home Based Telemedicine group and 94 patients in the Usual Care group. At baseline and at the end of the study, patients in both groups were seen in a cardiology office. Patients in Home Based Telemedicine group additionally were followed by a physician-nurse, through scheduled and unscheduled telephone appointments. These patients also received a blood pressure measuring device that could transmit the readings to a central data monitor via secure data connection.

**Results:**

During the study period (80 ± 25 days), a total of 17401 blood pressure measurements were taken in the Home Based Telemedicine group corresponding to 236 ± 136 readings per patient and a mean daily measurement of 3 ± 1.7. The scheduled telephone contacts (initiated by the nurse) equaled to 5.2 ± 4.3/patient (370 in total) and the unscheduled telephone contacts (initiated by the patients) were 0.4 ± 0.9/patient (30 in total). The mean systolic blood pressure values decreased from 153 ± 19 mmHg to 130 ± 15 mmHg (p < 0.0001) at the end of the study and diastolic blood pressure values decreased from 89 ± 10 mmHg to 76 ± 11 mmHg (p < 0.0001). In the Usual Care group, the mean systolic blood pressure values decreased from 156 ± 16 mmHg to 149 ± 17 mmHg (p < 0.05) at the end of the study and diastolic blood pressure values decreased from 90 ± 8 mmHg to 86 ± 9 mmHg (p < 0.05). The changes in drug therapy initiated following telephone contacts were 1.81 ± 1.73 per patient.

**Conclusions:**

The addition of a structured physician-nurse approach supported by remote telemonitoring of blood pressure is likely to improve outcome in patients with uncontrolled hypertension.

## Background

Many epidemiological studies have shown that the treatment and control of blood pressure (BP) is inadequate in more than 50% of hypertensive patients in spite of availability of several classes of well tolerated and effective antihypertensive drugs [1,2]. Several factors including the use of suboptimal doses of drugs, inadequate or ineffective treatments and poor drug compliance may be the reason for this phenomenon [3,4].

The guidelines of the European Society of Cardiology (ESC)/European Society of Hypertension (ESH) [5] recommend close collaboration between the general practitioners (GPs) and specialist centers for diagnosing and treating hypertensive patients. This closer collaboration results in better stabilization and maintenance of BP values in the reference range, over time [6-8].

A number of studies suggest the use of teleconsultation to facilitate the exchange of information and communication between GPs and specialists, as well as telemonitoring for the transmission of patient parameters, including BP [4,9-13]. The available data show that it is possible to improve BP control in hypertensive patients, either with or without complications, by changing treatment regimens via a telemedicine approach [4,7].

Systematic reviews have shown that in patients with hypertension, home blood pressure monitoring compared to a simple check at GPs offices may play an important role in improving control of systolic and diastolic BP (SBP and DBP) and treatment adjustment [9,14-17]. Published papers increasingly show the central and crucial role of properly trained nursing staff in coordinating all the health care professionals involved in the home management program, including providing advice to patients on life style changes, diet, exercise and smoking habits [18-20].

Reaching and maintaining the target value of blood pressure, the adherence to therapy, the reporting of the drug side effects and the change of life-style condition to reduce cardiovascular risk factors are the gold standard of our Home-Based Telemedicine (HBT) service.

The aim of the current non randomized “real life” study was to evaluate the effectiveness of a structured physician-nurse approach supported by remote BP telemonitoring, in patients with uncontrolled hypertension. The effectiveness was evaluated by assessing changes in BP values confirmed by ABPM. The data were compared with those collected in patients being followed in usual care conditions during the same period.

We also verified the costs of this service to provide credibility and evidence of its economic viability, so that this model could be applied to large number of patients in future studies.

## Methods

### Study design and participants

The study was approved by the Central Ethic Committee of Fondazione Salvatore Maugeri (Pavia, Italy) and followed the principles stated in the Declaration of Helsinki.

All patients were consecutively screened between September 2009 and September 2011 Patients came from a rural region and were referred to our outpatient clinic (at Fondazione Maugeri, Lumezzane Medical Center) by GPs for a cardiologic consultation.

The inclusion criteria were: i) new finding of BP values >140/90 mmHg or history of 24-h ambulatory BP monitoring (ABPM) values ≥125/80 according to ESC guidelines - ESH 2007 [21] or previous diagnosis of hypertension with evidence of BP values not adequately controlled by the current therapy; ii) age > 18 year and iii) having an in-home analog telephone line.

We excluded patients residing in long term care facilities, and those with severely cognitive impairments or those with poor prognosis.

All patients involved in the study were from a rural-mountain region, where cellular phone connection problems are quite frequent. Therefore, we first check the T-Mobile coverage maps of the patient’s zone; then asked the patient to confirm the availability of telephone coverage. For this reason we were obliged to enrol in the HBT group only patients who could prove to have a good cellular phone connection in their living area, while patients without cellular coverage were included in the Usual Care (UC) group.

Because of the nature of the intervention, neither the patients nor the physicians were blinded to the patient’s group assignment. However outcome assessors and data analysts were blinded.

### Procedures

All the patients were submitted to a cardiology office visit at baseline (T0) and at the end of the study (T1). During these visits, BP measurements were done always using the same oscillometric device following published guidelines [21]. The device measured the BP three times at intervals and the mean of three values was recorded as the BP for that visit. If needed, current therapy was changed or new antihypertensive drugs were started.

At T1, patients performed an ABPM using a validated Mobil O Graph (I.E.M. GMBH, Stolberg, DE) to confirm the documented BP values recorded at this visit. The duration of home follow-up was spread over a minimum of 40 days to a maximum of 120 days. The variability was related to the time required to reach the target value of BP. The BP monitoring was ended after at least 10 days of stable BP target values.

### HBT group

The service consisted of a structured physician-directed, nurse-managed telephone support added to a BP telemonitoring. The staff included hypertension specialists, cardiologists and trained nurses.

The structured telephone support has been described in previous publications [20,22]. In brief our approach included the following:

– Scheduled telephone appointments: these were done approximately every 15 days. During these contacts, the nurse carried out a standardized interview on general clinical condition of the patients. The patient was also counseled on weight management and physical activity, smoking cessation, dietary changes and stress management.

– The patients were asked information about their prescribed drugs and whether they took them regularly. If drug compliance was poor, the nurse offered strategies to improve patient’s compliance.

– Unscheduled appointments: these were initiated by the patient when they experienced signs and symptoms or had questions about their therapy. The patient had the opportunity to contact the nurse at any time of the day through the service centre and report any clinical problems.

Both during scheduled and unscheduled telephone appointments the nurse relayed relevant information to the clinical specialist and based on the available information the specialist provided feedback or modified the patient’s therapy if found necessary. European guidelines [21] were followed to titrate hypertensive drug with the aim to reach BP controlled values.

**Figure 1:**
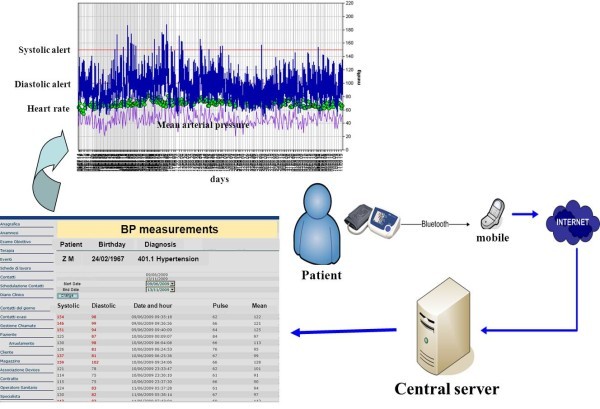
**Summary diagram of the home-based blood pressure telemonitoring system.** BP indicates blood pressure.

Patients in this group were supplied with a blood pressure measuring device (model A&D UA-767 Plus BT) and a mobile phone which had a Java 2 Platform Micro Edition (J2ME) application installed. The A&D sensor could transmit the BP values to a mobile through Bluetooth connection and it could send them to a server via secure data connection. At the beginning of the study an educational meeting was held where the nurse explained the study to the patients and taught them the correct way to use the home BP instruments and the mobile phone and the correct way to measure BP based on the published guidelines [21]. Figure 1 shows a summary diagram of the system.

The nurse, by entering the appropriate passwords within the web platform, could retrieve, under the “personal health record” of the patient, the BP values which were automatically updated with out-of-range values highlighted. In case of abnormal readings, the nurse could contact the patient through a service centre. The nurse could also verify that the patient measured their BP with the agreed frequency. In case of no measurement for at least three days, the nurse contacted the patient.

### UC group

In this group the patients were followed by their GPs without any structured program.

### Procedure to evaluate costs of HBT service

The costs were evaluated as follows:

1. *Fixed costs* included device rentals, equipment purchases plus installation, installation of telecommunication lines, monthly line charges, and maintenance costs;

2. *Variable costs* included expenses for service centre services, nurse-tutors, second opinions and telecommunication.

The average annual salaries for nurse and specialist were obtained and were used to compute the total cost of health service staff, which included costs for duration of consultations and costs for the extra-head activity such as time for enrolment, discharge, web chart use, and administrative activities. The service center costs included the license for using the web platform to carry on the service including the patient’s personal health record, the call center calls and the general costs as service etc.…

### Statistics

The analysis was carried out with Graph Pad Prism 4 version 4.03 and MedCalc version 11.4.2. Statistical significance was assessed by using a paired *t*-test for two samples with equal variance and with 2-sided distribution of the two treatment arms and 2-way ANOVA performed within-subject factors (SBP and DBP at T0 and T1 between groups). The χ-square analysis and analysis by Fisher’s exact test was carried out. A p-value <0.05 was considered statistically significant. Data are represented as mean ± standard deviation (SD) and as mean ± 95% Confidence Interval.

## Results

### Patient population

**Figure 2:**
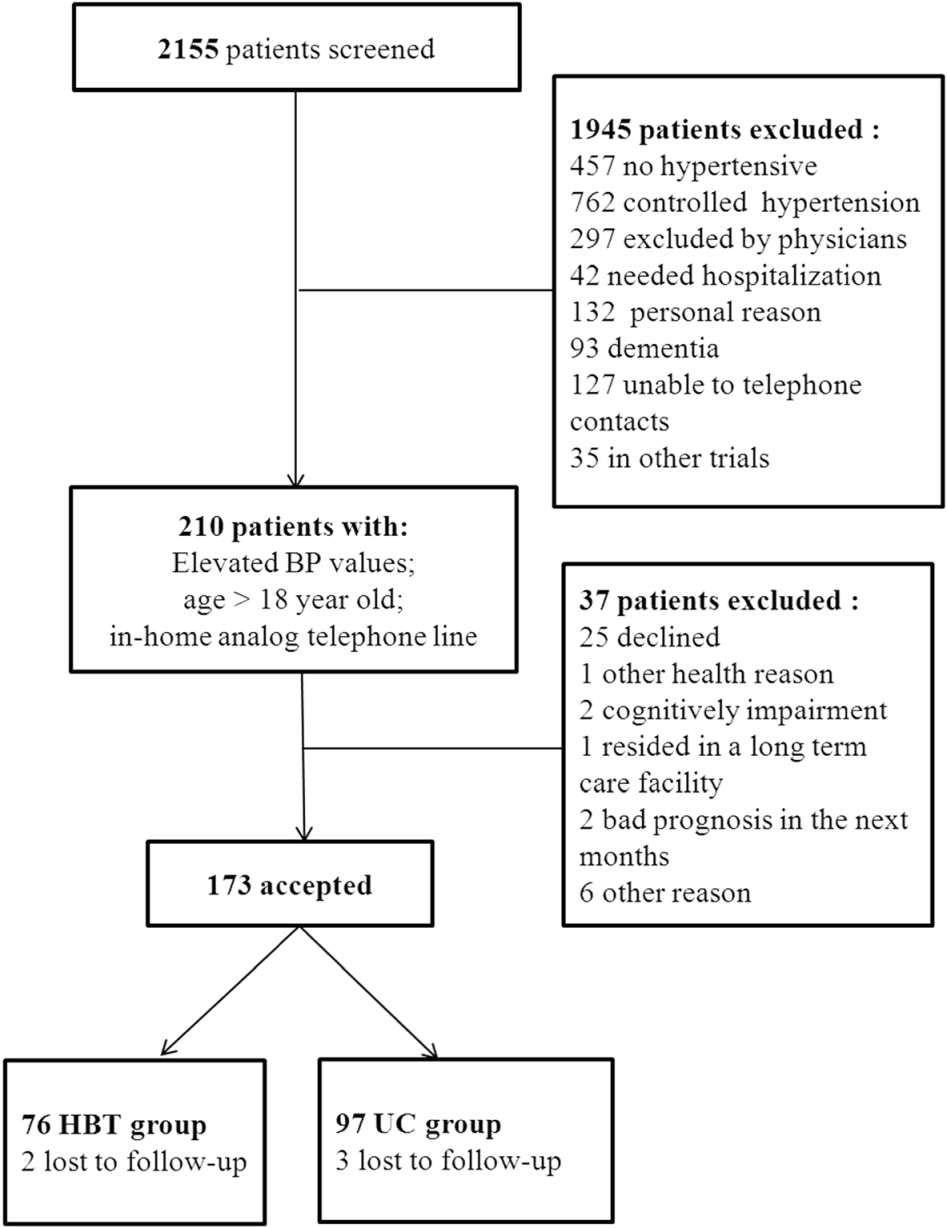
**Participants flow.** HBT, indicates home-based telemedicine; UC, indicates usual care.

Figure 2 shows participants flow throughout the study. From 2155 patients screened, 210 patients (10%) had met inclusion criteria. 37 patients were excluded. 74 patients (38 males and 36 females) were enrolled in the HBT group and 94 patients (49 males and 45 females) in the UC group.

Table 1 shows the characteristics of the patient population at baseline (T0). No significant differences in the main parameters were found between the two groups.

**Table 1:** Characteristics of the two patient populations at baseline (T0)

**Patients**	**HBT group**	**UC group**	**P=**
	**n = 74**	**n = 94**	
M/F (%)	38/36 (51/49%)	50/44 (53/47%)	0.77
mean age ± SD (years)	59.7 ± 12.5	59.1 ± 13.3	0.76
Mean follow-up (days)	80 ± 25	82 ± 28	0.54
Pts followed for 40–65 days	21	29	
Pts followed for 66–99 days	38	33	
Pts followed for 100–120 days	10	25	
Pts followed for 120–130 days	5	7	
***Diagnosis:***			
Hypertension (H)	74 (100%)	94 (100%)	
H + hypertensive cardiomyopathy	10 (14%)	9 (10%)	
H + ischemic cardiomyopathy	1 (1.4%)	2 (2%)	
BMI	27 ± 6	28 ± 5.2	0.25
***Risk factors (%):***			0.45
Diabetes	12 (16%)	18 (19%)	
Obesity	21 (28%)	30 (32%)	
Family History	66 (89%)	77 (82%)	
Dyslipidemia	20 (27%)	29 (31%)	
Smokers	12 (16%)	10 (11%)	
Ex-smokers	13 (18%)	8 (9%)	
Patients with >2 risks	43 (58%)	50 (53%)	

BMI indicates body mass index; HBT, indicates home-based telemedicine; UC, indicates usual care; SD, standard deviation.

### Telemedicine service

During the period (80 ± 25 days), 17401 BP measurements were taken (236 ± 136/patient) corresponding to a mean daily measurement of 3 ± 1.7. The average number of BP measurements per day was 5.6 ± 2.5 during the first week and 4.3 ± 3.8 measurements/day during the last week of the study.

The telephone contacts initiated by the nurse during the study equaled to 5.2 ± 4.3/patient (370 in total). All actions were focused on educational reinforcements and therapeutic compliance verifications. The changes in drug therapy suggested during the telephone contacts were 1.81 ± 1.73 per patient.

The contacts initiated by the patients were 0.4 ± 0.9/patient (30 in total).

Table 2 shows the data relevant to the home telemedicine intervention.

**Table 2:** Structured telephone support

**Scheduled contacts per patient (total)**	**5.2 ± 4.3 (370)**
**Reasons for scheduled contacts:**	
- scheduled control	148
- BP values above the threshold	176
- BP values below the threshold	11
- data not sent	35
**Actions undertaken during scheduled contacts:**	
- educational reinforcements., therapeutic compliance verifications, rescheduled contacts, etc.…	370
- therapeutic changes	124
- contacts with cardiologist	8
- contacts with other specialists	2
- requests for diagnostic examinations	7
- sending to the emergency room for syncope	1
**Unscheduled contacts per patient (total)**	**0.4 ± 0.9 (30)**
**Reasons for unscheduled contacts:**	
- communications	5
- BP values above the threshold	18
- BP values below the threshold	4
- comfort request for anxiety	3
**Actions undertaken:**	
- educational reinforcements., therapeutic compliance verifications, rescheduled contacts	27
- therapeutic changes	10
- contacts with cardiologist	2
- contacts with GP	1

BP indicates blood pressure; GP, general practitioner.

### BP measurements

**Figure 3:**
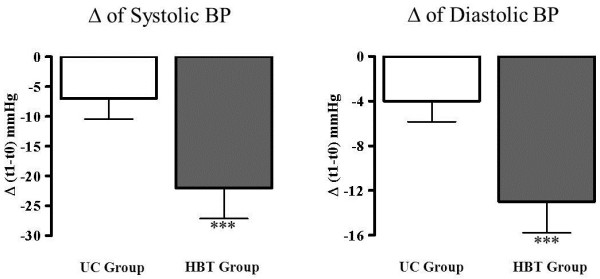
**Variation of systolic BP and diastolic BP of the two groups of patients measured during the study period.** BP indicates blood pressure, HBT, indicates home-based telemedicine, UC, indicates usual care ***p < 0.0001 within group.

Figure 3 shows SBP and DBP changes in the two groups. No significant differences in BP values were observed between the two groups at baseline (T0). In the UC group, at the end of the study period, the mean SBP values decreased from 156 ± 16 mmHg to 149 ± 17 mmHg (-6.8 ± 14.6, p < 0.05) and mean DBP values from 90 ± 8 mmHg to 86 ± 9 mmHg (-4 ± 8, p < 0.05).

In the HBT group, the mean SBP values decreased from 153 ± 19 mmHg to 130 ± 15 mmHg (-22.3 ± 22.6 mmHg, p < 0.0001) and DBP values decreased from 89 ± 10 mmHg to 76 ± 11 mmHg (-13 ± 12,2 mmHg, p < 0.0001). The ABPM values, at the end of the study (T1) were as follows: SBP was 136 ± 11 mmHg in the UC group and 125 ± 11 mmHg in the HBT group; DBP was 81 ± 9 mmHg in the UC group and 76 ± 8 mmHg in the HBT group.

BP values measured at the office at final visit in the UC group vs. the values measured at the baseline showed poor BP control with 81% of patients with out-of-range SBP values and 62% of patients with out-of-range DBP values. On the contrary, in HBT group the percentage of patients with out-of-range values at the final visit were 26% for SBP and 8% for DBP.

The 2-way ANOVA performed on BP values of UC group vs. HBT Group confirmed the better BP control in the HBP group for both SBP and DBP (p < 0.001).

### Drugs used during the study

The analysis by Fisher’s exact test carried out between treatment groups at T0 and T1 showed a significant increase in the use of Angiotensin Converting Enzyme -inhibitor and/or Angiotensin II antagonists (p = 0.001), diuretic (p < 0.05) in patients in the HBT group compared with the UC group. The use of α-adrenergic drugs in HBT group was increased but the increase was not statistically significant.

At the final visit, the mean number of drugs prescribed per patient was greater in the HBT group (2.5 ± 1.1 drugs/patient, with T1 vs. T0 p <0.0001) compared with the UC group (2.0 ± 1.1 drugs/patient, with non-significant T1 vs. T0). In fact, we observed that in the UC group only 23% of patients had their drug regimen changed compared to 70% in the HBT group.

#### Cost was calculated as follows

i) Rental of BP device (A&D UA-767BT) and mobile phone software and data connection = 76 €/patient

ii) Service Centre expenses = 30 €/patient

iii) Reimbursement for nurse-tutor = 45 €/patient

iv) Physician specialist costs = 15 €/patient [specialist’s intervention only during telemedicine, more or less 10 minute for patient].

Total costs = 166 €/patient (2.1 €/patient/day)

## Discussion

The results obtained by this telemedicine approach in patients with poor control of hypertension would seem to confirm the importance of a much higher level of compliance with respect to regular BP measurement, drug compliance and therapy adjustments [23,24]. Technology alone will likely fail to increase patient’s self-management goals and to improve patient’ outcomes. Thus, patients with out-of-range BP may benefit from more intensive intervention that can be provided by nurses in a structured model connected with physicians.

In comparison with the previous studies, the educational program run by the nurses is fundamental; the physicians are immediately made aware when BP values are outside the range. This leads to re-evaluation of drug compliance or drug therapy. Moreover, when the patients realize that their adherence is constantly and carefully checked, they acquire a feeling of greater control and consequently higher compliance which contributes to positive effects that can be observed in the first few weeks.

Through telemedicine, symptoms of hypertension or hypotension, malaise, or requests for comfort or simple communications needs can be relayed in real time leading to greater patient satisfaction. Moreover, the continuous monitoring of patients followed by appropriate intervention is a critical attribute of the program. This is probably the reason why in the HBT group there was a higher number of patients (70%) in whom the drug therapy was modified compared to those in the UC group (23%). The complete optimization of the treatment probably requires a longer period with more frequent nurse-patient contacts. In this respect, telemedicine could be an excellent addition which could reduce the number of office visits [12].

It is true that in both groups, at final visit, both SBP and DBP were significantly lower but only in the HBT group there was a clinically relevant BP reduction, with values overlapping the BP levels which are recognized as the target according to International Guidelines [21].

A number of meta-analysis studies have shown that a BP monitoring, compared to a single office visit measurement, has the potential to better manage uncontrolled hypertension [9,14-17].

We have obtained positive results thanks to the use of a well-organized telemedicine program in which nursing and specialist medical staff play an important role [8,22,25]. Another important factor was that the technique was easy to use and was reliable with no major complaints from the patients.

BP is usually sub-optimally controlled due to various factors including the fact that it is often asymptomatic, requires continuous treatment for life, with drug related side effects leading to poor drug compliance [26-28]. The importance of self BP measurement is an educational goal for the patient. This goal cannot be achieved by a single office visit. Furthermore, because of the issues with cost containment, repeated office visits are discouraged. In addition, the Telemedicine approach which combines nurse support plus remote monitoring, reduces the problem of “white coat syndrome” [29]. An effective monitoring program for a period of 2–4 months associated with a structured physician-nurse approach can lead to beneficial overall effect for patients with uncontrolled hypertension.

Telemedicine services provide an additional value to standard of care with only a small cost addition. In view of the results obtained (greater significant reduction in the HBT group vs. the SC group), it would be crucial to plan randomized studies with a longer home follow-up to confirm these findings and to verify the efficacy of Telemedicine intervention in the reduction of acute cardiovascular events, which are known to increase by 30% for every 10 mmHg increase in BP [21].

### Limitation of the study

One limitation of the study is that it was carried out following a non-randomized design.

Another limitation is the lack of baseline ABPM since the study was conducted in the real clinical practice where ABPM is not always performed.

The cost analysis considers only the services provided to the patient in the HBT group and does not evaluate the cost effectiveness of the service.

## Conclusions

This study indicates that a telemedicine service, consisting of structured physician-directed, nurse-managed telephone support added to remote telemonitoring is feasible, reliable and has a positive effect on blood pressure control. This type of approach could decrease the number of hypertensive patients that are currently being treated but who are still uncontrolled.

## Abbreviations

HBT: Home-based telemedicine; UC: Usual care; BP: Blood pressure; SBP: Systolic blood pressure; DBP: Diastolic blood pressure; ABPM: 24-h ambulatory BP monitoring; ESC: European Society of Cardiology; ESH: European Society of Hypertension.

## Competing interests

The authors declare that they have no competing interests.

## Authors’ contributions

PB, SS, FB, FR, MLM contributed to the study design; PB, SS drafted the manuscript; PB performed data interpretation and statistical analysis, FB, FR contributed to acquisition of data; All authors reviewed and approved the final manuscript.

## Pre-publication history

The pre-publication history for this paper can be accessed here:

http://www.biomedcentral.com/1472-6947/14/52/prepub
